# Optimization of Fungal Dextranase Production and Its Antibiofilm Activity, Encapsulation and Stability in Toothpaste

**DOI:** 10.3390/molecules25204784

**Published:** 2020-10-18

**Authors:** Nucharee Juntarachot, Duangporn Kantachote, Sartjin Peerajan, Sasithorn Sirilun, Chaiyavat Chaiyasut

**Affiliations:** 1 Innovation Center for Holistic Health, Nutraceuticals and Cosmeceuticals, Faculty of Pharmacy, Chiang Mai University, Chiang Mai 50200, Thailand; nucharee_t@cmu.ac.th; 2 Department of Microbiology, Division of Biological Science, Faculty of Science, Prince of Songkla University, Hat Yai 90112, Thailand; duangporn.k@psu.ac.th; 3 Health Innovation Institute, Chiang Mai 50200, Thailand; s.peerajan@gmail.com

**Keywords:** alginate beads, antiplaque, Box–Behnken design, dextran, *Penicillium roquefortii*, Plackett–Burman design, *Streptococcus mutans*

## Abstract

Dextranase catalyzes the degradation of the substrate dextran, which is a component of plaque biofilm. This enzyme is involved in antiplaque accumulation, which can prevent dental caries. The activity of crude dextranase from *Penicillium roquefortii* TISTR 3511 was assessed, and the maximum value (7.61 unit/g) was obtained at 37 °C and pH 6. The Plackett–Burman design was used to obtain significant factors for enhancing fungal dextranase production, and three influencing factors were found: Dextran, yeast extract concentration and inoculum age. Subsequently, the significant factors were optimized with the Box–Behnken design, and the most suitable condition for dextranase activity at 30.24 unit/g was achieved with 80 g/L dextran, 30 g/L yeast extract and five day- old inoculum. The use of 0.85% alginate beads for encapsulation exhibited maximum dextranase activity at 25.18 unit/g beads, and this activity was stable in toothpaste for three months of testing. This study explored the potential production of fungal dextranase under optimal conditions and its encapsulation using alginate for the possibility of applying encapsulated dextranase as an additive in toothpaste products for preventing dental caries.

## 1. Introduction

Dextranase is an enzyme that catalyzes the endohydrolysis of α-(1-6)-d-glycoside linkages in dextran [1]. Dental plaque or biofilm in the oral cavity contains dextran, which is a product of dental caries pathogens such as *Streptococcus mutans* and *Streptococcus sobrinus* [2,3,4]. It is well recognized that dextranase breaks down the structure of biofilm [5]. In recent years, dextranase has received considerable attention in the food, medicine, and dental fields [6]. Currently, there are many active ingredients used in different formulations to prevent tooth decay as well. Consequently, the application of dextranase in oral care is an emerging trend in the world as dextranases can be added in toothpaste or other dental care products to prevent dental caries [1].

Dextranase has been found in plants, mammalian tissues, fungi and bacteria; however, fungi are a rich source of this enzyme [7]. Dextranase activity has been found in a wide variety of fungi, such as *Aspergillus niger*, *Chaetomium gracile* and *Penicillium* species [8]. Fungal dextranase is widely used in the food industry as it is safe and easy to harvest [9]. Thus, fungi are the most important commercial dextranase producer [10]. Commercial fungal dextranases are applied to maintain food safety and extend the shelf life of foods [11].

To achieve the highest production of any metabolites from microorganisms, optimal conditions are required. Plackett–Burman (PB) design is the most popular approach to study optimization as it is a primary screening of variables that rely on the yield of a target metabolite [12]. To enhance the production of the metabolites, i.e., dextranase, the three most significant factors positively affecting the production were selected according to the PB experiment and then they were optimized by employing Box–Behnken (BB) design [13]. The BB design has been accepted as a good design for the optimization of key variables [14]. The BB design presents the optimum conditions for the success of the best response through a relatively small number of experiments [15].

Dextranase has been applied in many industries; however, it has limited use due to poor stability in harsh environments, which has an adverse effect on enzyme activity [6]. The causes of instability of the enzyme in oral care products are many, such as temperature, acid, base, surfactant and other components of oral care products [16]. Therefore, it is highly desirable to develop effective methods for increasing the stability of dextranase for application in oral care products [6]. Encapsulation is commonly used to protect active compounds against adverse environmental and processing conditions or to provide controlled release in processed foods [17]. After encapsulation, the active compounds are significantly improved, indicating the potential application in active packaging [18]. Hence, it is possible to apply encapsulation of any enzymes in industrial and cosmetic products [1]. Many biopolymers are used for encapsulation; however, alginate is one of the most frequently used due to the following reasons: Mild gelling property, non-toxicity, inexpensive and versatile method for encapsulation of enzymes [19].

Regarding the above information, this research aims to determine the optimal condition for producing dextranase based on optimization by acceptable designs. The optimal condition to encapsulate dextranase in the alginate matrix was also investigated to obtain an encapsulated enzyme with higher stability for possible application in oral care products.

## 2. Results

### 2.1. Fungal Dextranase Production

**Figure A1:**
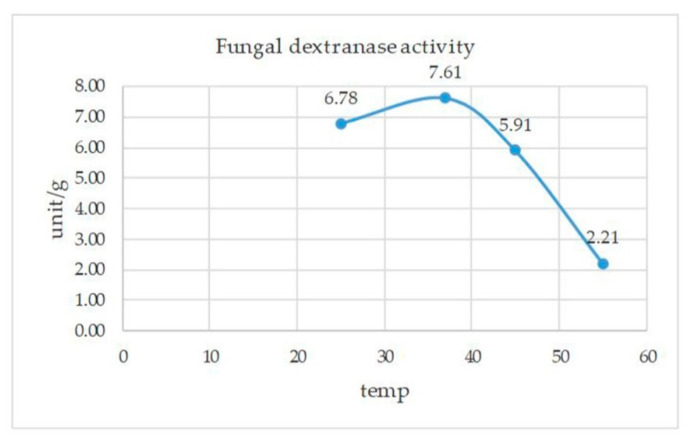
The suitable condition of fungal dextranase activity was 7.61 unit/g at pH of 6 and 37 °C.

One percent (1%) dextran was the substrate in a modified medium and it was found that *P*. *roquefortii* TISTR 3511 was able to produce dextranase. However, *Aspergillus niger* TISTR 3063 did not show dextranase activity. The crude dextranase activity of strain TISTR 3511 was examined for its optimal pH and temperature. The highest dextranase enzyme activity, 5.91 unit/g, was observed at 30 °C, pH of 6, followed by pH of 7 and 5, while the minimum was observed at pH of 3–4. The optimal temperature of this fungal dextranase activity under the optimal pH 6 was between 25 and 45 °C. A big decrease was observed at 55 °C. For the overall result, the highest activity of crude dextranase from strain TISTR 3511 was 7.61 unit/g at pH of 6 and 37 °C (Appendix A, Figure A1). Conditions suitable for fungal dextranase activity were selected to culture the fungus and measure its activity in all experiments of this research.

### 2.2. Effect of Crude Dextranase on Biofilm formation of s. mutans

The effect of sub-mic (1/2mic; 0.71 unit/g) of the crude dextranase on biofilm formation was investigated using the method described by sato et al. (2018). The experiment performed in our study enabled us to measure the biofilm formation of the tested bacteria, *S. mutans* atcc 25175 and subsequently the rate of adherence. The biofilm from *S. mutans* in the presence of crude dextranase from *P. roquefortii* tistr 3511 was moderately adherent compared with strongly adherent in the positive control with no addition of dextranase (Table 1).

### 2.3. Antibiofilm Assay: Confocal Laser Scanning Microscopy (CLSM)

**Figure 1:**
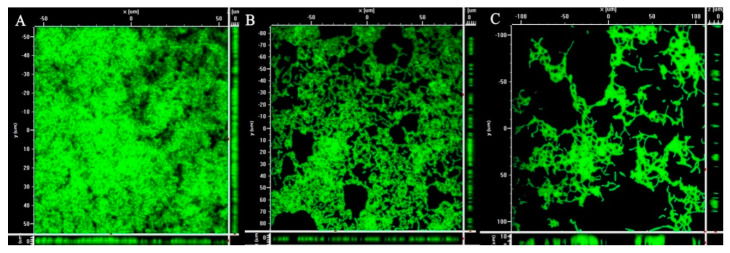
Biofilm analysis using confocal laser scanning microscopy: (**A**) Representative image of biofilm formed by *S. mutans* ATCC25175, (**B**) biofilm in the presence of crude dextranase from *Penicillium roquefortii* TISTR 3511, (**C**) biofilm in the presence of commercial fungal dextranase.

As confocal Laser Scanning Microscopy (CLSM) is a popular method to study biofilm structure, it was carried out to confirm if the synergized effect of dextranase activity can inhibit biofilm produced by *S*. *mutans* ATCC 25175. After 24 h incution, biofilm formation in the presence of the crude fungal dextranase from *P*. *roquefortii* TISTR 3511 was analyzed by CLSM after staining with SYTO 9 green fluorescents to evaluate the antibiofilm activity by comparing with controls (commercial dextranase and without dextranase). The staining is used to indicate cells including biofilm on the basis of the amount of biofilm formation [20,21]. The crude dextranase from *P*. *roquefortii* TISTR 3511 showed reduction in biofilm formation compared with the control with no addition of dextranase, which showed the presence of a dense biofilm produced by *S*. *mutans* ATCC 25175. (Figure 1). However, its antibiofilm ability was less than for commercial fungal dextranase. The representative images of Figure 1 obtained from at least three independent experiments that showed the similar images.

### 2.4. Optimization of Dextranase Production by Fungus

Plackett–Burman (PB) design has been proved to be the most effective method in optimizing the medium composition and cultivation for enzyme production, so this design was firstly used to determine the likely effects of nine possible factors including medium composition and cultivation on dextranase production as shown in Table 2.

Dextranase activity (Y) = 9.351 + 5.71A + 2.12B − 0.037C − 0.713D − 0.138E + 0.875F − 1.174G − 0.678H + 1.872I

where Y is a predicted response that depends on: A—dextran; B—yeast extract; C—K_2_HPO_4_; D—NaNO_3_; E—MgSO_4_.7H2O; F—incubation time; G—inoculum size; H—medium volume and I—inoculum age. The significant factors were further studied using BB design to optimize the level of each for dextranase production by *Penicillium roquefortii* TISTR 3511.

The Box–Behnken design was secondly used to optimize the levels of those three significant factors to improve dextranase production. This design also studied the relationship between parameters and the dextranase production of *P. roquefortii* TISTR 3511. Table 3 shows that experimental run number 14 produced the highest activity of the enzyme. The optimum conditions were 80 g/L dextran, 30 g/L yeast extract and five days inoculum age, which had the highest dextranase activity (30.24 unit/g.). Run number 14 was the most suitable condition for producing dextranase enzyme from *P. roquefortii* TISTR 3511.

Table 4 shows that dextran, yeast extract and inoculum age had positive effects on the production of dextranase (*p* < 0.05). A smaller magnitude of *p*-value indicates a more significant correlation with the corresponding coefficient. This table also found that, regarding the lack of fit, the F value was not significant, with an F value of 2.65 and a *p*-value of 0.185. That means that the model was valid for further analysis [22]. The quadratic model was found to yield the best fit of R^2^, adjusted R^2^ and predicted R^2^ values of 0.969, 0.929 and 0.655, respectively. In summary, the quadratic model was suitable to explain the association between the significant factors and dextranase activity. With multiple regression analysis on the experimental data, a predictive response for dextranase activity could be obtained via the second-order polynomial equation, as follows:

Dextranase activity (Y) = 28.262 + 1.543A + 2.039B + 0.704C + 0.315AB + 0.475AC − 0.053BC − 1.442AA − 0.840BB − 0.800CC

Y is the response (dextranase enzyme activity) and A (dextranase), B (yeast extract) and C (inoculum age) are the coded values of the independent variables. The ANOVA of the regression model demonstrates that the model is highly significant (*p* < 0.001). The conditions that produced the highest dextranase activity (30.24 unit/g) were 80 g/L dextran, 30 g/L yeast extract and five days of inoculum age. Hence, suitable conditions were used to produce the dextranase by *P. roquefortii* TISTR 3511 for further studies.

**Figure 2:**
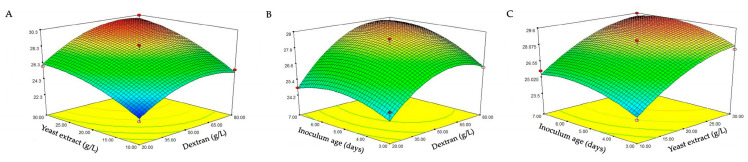
Response surface plots for the interactions between the independent variables. (**A**): Effect of yeast extract (g/L) and dextran (g/L); (**B**): Effect of inoculum age (days) and dextran (g/L); (**C**): Effect of inoculum age (days) and yeast extract (g/L). The values in the figure indicated the level of dextranase enzyme activity (unit/g).

Figure 2 shows the response surface plots for the interactions between various factors, where red and blue are the maximum and minimum dextranase enzyme activities of encapsulation, respectively. The interaction between the dextran (g/L) and yeast extract (g/L) is shown in Figure 2A. The higher levels of both factors caused a higher dextranase activity, while the interaction between dextran (g/L) and inoculum age (days) (Figure 2B) suggests that dextranase activity depended on the amount of dextran, particularly at 80 g/L and with an inoculum age in the range of 5–7 days. The interaction between yeast extract (g/L) and inoculum age (Figure 2C) was similar to the interaction of dextran and inoculum age.

Considering the responses (trapped dextranase activity in alginate beads), it was found that the optimum condition was the same as 80 g/L dextran, 30 g/L yeast extract and five days of inoculum age. Therefore, this condition was selected for producing dextranase for further study.

### 2.5. The Encapsulation of Dextranase from P. roquefortii TISTR 3511

**Figure 3:**
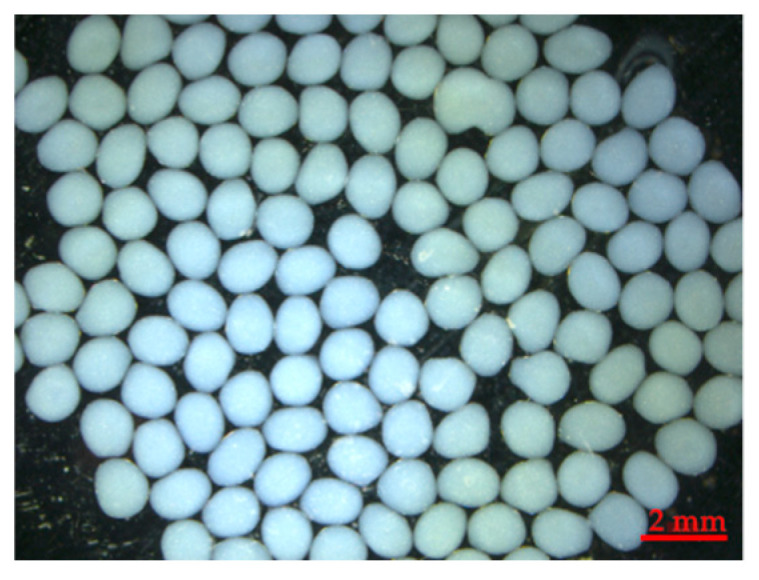
The encapsulation of crude dextranase from *Penicillium roquefortii* TISTR 3511 in alginate beads.

Crude dextranase from strain TISTR 3511 was encapsulated under a suitable condition in alginate matrix at pH of 7, 20% *w*/*v* calcium chloride and 0.85% *w*/*v* sodium alginate. Figure 3 shows the regular spherical shape of alginate beads containing encapsulated fungal dextranase from *P. roquefortii* TISTR 3511. The average diameter of 30 beads was 1.34 ± 0.02 mm. Dextranase activity in alginate beads was observed to be 25.18 unit/g beads.

### 2.6. The Stability of Dextranase Activity in a Toothpaste Base

The stability of encapsulated dextranase alginate beads in toothpaste formulation was investigated at baseline and after three-month storage. No significant difference (*p* = 0.090) was found in encapsulated dextranase activity between the initial time and after storage of the products at 40 ± 2 °C for three months (Table 5). An overview of the stability of the toothpaste according to the good properties, such as color, odor, viscosity, pH and homogeneity, found no change in its characteristics after three months compared to the baseline. The results indicate that the dextranase activity was stable after storage of the products at recommended temperature for testing stability for three months.

## 3. Discussion

Dextranase is produced by various microorganisms, such as yeasts, bacteria and fungi; however, commercial dextranases are commonly produced from a fungal source. This is due to the “generally recognized as safe” (GRAS) status of fungal dextranases and their higher activity and yield [23]. For example, *Chaetomium gracile* and *Penicillium* spp. are good candidates for producing dextranase on a commercial scale [8,24]. Recently, dextranase has attracted significant commercial interest in cosmetics, drug formulations and oral care products [19]. Hence, the present study investigated the possibility of producing fungal dextranases for application in oral care products, like toothpaste. Nevertheless, it was found that only *P. roquefortii* TISTR 3511 was able to produce dextranase, while *A. niger* TISTR 3063 was unable to produce this enzyme in the conditions tested. 

This study found that *P. roquefortii* TISTR 3511 is an efficient dextranase producer. Interestingly, this fungal dextranase as a crude enzyme at sub-MIC showed a significant effect in reducing adherent capability from *S. mutans* ATCC 25175 (Table 1). This suggests that biofilm formation was significantly reduced in the presence of dextranase compared to the positive control with no dextranase. This was due to the biofilm produced from *S. mutans* playing an important role in adhering to the surface [25]. This means that dextran produced by *S. mutans* was strongly degraded by dextranase, indicating that the crude dextranase acts as an anti-adhesion property as a defense mechanism to disturb biofilm formation by *S. mutans* (Table 1). This was confirmed by the evidence of the loose biofilm in the presence of fungal dextranases from TISTR 3511 compared with the dense biofilm in the absence of dextranase (Figure 1). This indicates that crude dextranase from *P. roquefortii* TISTR 3511 reduced cell attachment as no dextran or less dextran to form biofilm. *S. mutans* is the main cause of dental decay in human teeth and a key modulator of the development of cariogenic biofilms [26]. This confirms a previous finding regarding the role of dextranase as a possible alternative compound against *S. mutans* biofilm formation by degrading dextran as a main cause of dental plaques. Hence, it could be possible to use this fungal dextranase for preventing dental caries [27]. For this reason, the use of this fungal dextranase to treat dental caries has attracted a great deal of attention, particularly for the degradation of dextran in dental plaques, and this has led to the promotion of fungal dextranase production from strain TISTR 3511.

Many factors affect the production of the dextranase enzyme, such as the strain of microorganism, culture medium and fermentation conditions; therefore, the optimization technique plays a significant role in the improvement of production. The factors affecting dextranase production were determined using the PB design, which has been proved to be the most effective process in screening factors for optimizing enzyme production [12]. Then, the most significant factors were further studied using the BB design, which is an appropriate and modern optimization method [15]. This is confirmed by the results of the present study, which showed that dextran, yeast extract and age of inoculum are significant factors that influence fungal dextranase production (Table 2) since, under its suitable conditions (pH 6 and 37 °C) (Appendix A Figure A1 and Table 3), a remarkable increase in dextranase activity from 7.61 unit/g to 30.24 unit/g occurred, roughly a four-fold increase (30.24/7.61 = 3.97). The suitable conditions for producing dextranase enzyme (unit/g) from *P. roquefortii* TISTR 3511 were conditions 1, 14 and 17, which showed dextranase activities of 29.57, 30.24 and 28.47 unit/g, respectively. These dextranase activities were found to be statistically different to each other using ANOVA. The results show that activity numbers 14 and 17 (30.24 ± 0.32 and 28.47 ± 0.16 unit/g,) were significantly different at *p*-value 0.001, while there was no significant difference for activity numbers 14 and 1 (30.24 ± 0.32 and 29.57 ± 1.52 unit/g,) at *p*-value 0.168. However, the conditions of 14 were better than the conditions of 1 in producing the dextranase enzyme, as the culture time was shorter than for condition 1, which directly led to a significant increase in the cost of operation for condition 1.

To maintain the stability of dextranase activity for application in toothpaste, encapsulation is considered as a suitable procedure to preserve the substance; it protects the substance from adverse conditions [28]. Optimization of encapsulation for protecting dextranase is a very important stage. In the present study, a suitable model of encapsulation with food-grade fungal dextranase was used to encapsulate crude fungal dextranase from strain TISTR 3511. This experiment showed successful encapsulation of crude fungal dextranase in the alginate matrix, which is simple and very effective for the stability of encapsulated dextranase alginate beads in toothpaste after three-month storage (Table 5). It should be noted that the encapsulated dextranase activity (25.18 unit/g) decreased by approximately 16.73% compared with the unencapsulated enzyme (30.24 unit/g). This indicates that the optimal conditions for encapsulation still caused a decrease in dextranase activity; this is the main reason to support the choice of experimental run no. 14 for dextranase production (Table 3). Another reason might be incomplete release of encapsulated dextranase, and this should be further investigated to obtain higher efficiency of dextranase release from alginate beads. The dextranase activity in alginate beads was observed to be 25.18 unit/g beads. However, the activity was checked again before storage. The result showed that the activity was changed after the beads were mixed in toothpaste (T0) (21.69 unit/g beads). This is because toothpaste consists of surfactant, and the surface of the beads was in direct contact with surfactant and other ingredients of toothpaste, leading to some loss activity of dextranase in beads. This is the main reason why it is required to encapsulate dextranase in toothpaste for protecting its activity in harsh conditions. It should be noted that a higher release of dextranase from beads should be possible in case of tooth brushing due to the mechanical force during brushing. It is noted that the stability of the dextranase encapsulation after long-term storage for three month found no significant change. This suggests that the encapsulated dextranase in alginate beads was protected and it might be possible to apply this in developed toothpaste for preventing dental caries. This research explored an alternative health product for oral care by applying encapsulated dextranase in toothpaste. However, the safety of the dextranase enzyme from *P. roquefortii* TISTR 3511 must be evaluated before it becomes a new source of dextranase that is safe for human use. Further investigation on its potential as a candidate fungal dextranase to be applied in developing toothpaste to prevent dental caries is required.

## 4. Materials and Methods 

### 4.1. Fungal Dextranase Production

In this study, *Aspergillus niger* TISTR 3063 and *Penicillium roquefortii* TISTR 3511 were purchased from a culture collection, Thailand Institute of Scientific and Technological Research (TISTR), and they were used to produce crude dextranase. Each mold was cultured in broth medium that consisted of 1% dextran, 0.05% K_2_HPO_4_, 0.2% Yeast extract, 0.2% NaNO_3_, 0.2% MgSO_4_ ·7H2O and pH of 6 [29]. The mold was incubated at 30 °C, using a rotational speed of 120 rpm for 7 days. The fungal broth was filtered to remove mycelia, and its supernatant was used to determine dextranase activity. A commercial fungal dextranase from *Chaetomium gracile* (Mitsubishi chemical foods corporation, Japan) was also compared with the tested fungal strain based on anti-biofilm assay.

### 4.2. Measurement of Dextranase Activity

Dextranase activity was measured using an increasing ratio of reducing sugar concentration in the reaction with 3,5-dinitrosalicylic acid reagent [1]. A mixture of 125 µL of crude dextranase from fungus and 125 µL of dextran solution (20 mg/mL) was incubated at 37 °C for 30 min. The reaction was stopped after 30 min by transferring 250 µL aliquots of the enzyme-substrate mix into tubes containing 3,5-dinitrosalicylic acid reagent. The test tubes were immersed in a boiling water bath for 15 min until the color changed. The absorbance of the mixture was measured at 540 nm. One unit of dextranase activity (mmol maltose/min) was defined as the activity of the enzyme that catalyzed the liberation of 1 mmol of maltose in 1 min from dextran. In order to assess the possibility of adding crude dextranase in toothpaste to prevent dental caries, the optimal condition of this enzyme was investigated by varying pH (3, 4, 5, 6 and 7) and temperature (25, 37, 45 and 55 °C).

### 4.3. Effect of Crude Dextranase on Biofilm Formation of S. mutans

*P. roquefortii* TISTR 3511 was selected in this study due to its dextranase activity compared with no activity of *A. niger* TISTR 306. Before the study, Minimum Inhibitory Concentration (MIC) was determined in broth dilution. In brief, *S. mutans* ATCC 25175 was cultured in Tryptic Soy Broth (TSB) and adjusted to obtain 10^6^ cells/mL by normal saline solution. Then it was mixed with the serially diluted test of dextranase (144-0.07 unit/g concentrations) in a 96 well-plate and incubated at 37 °C, 5% CO_2_ for 24 h. The MIC was determined based on the growth of *S. mutans* measurement at 600 nm [30], and the MIC of dextranase from TISTR 3511 was titer 1:8. The effect of Sub-MIC (1/2 MIC) by crude dextranase from *P. roquefortii* TISTR 3511 against the biofilm formation of *S. mutans* was tested with the modification described by Sato et al. [31]. A 100 µL crude dextranase was added into 100 µL TSB containing the *S. mutans* ATCC 25175 cell suspension at 10^6^ CFU/mL. Uninoculated medium and bacterial cultivation in the absence of dextranase were served as negative and positive controls, respectively. All tests were carried out three different times, and the results were averaged. The 96 well plates were incubated at 37 °C, 5% CO_2_ for 24 h. After incubation, biofilm was stained with 0.4% crystal violet for 15 min. The cells were then washed three times with sterile distilled water and air-dried for 60 min. Stained biofilm cells were de-stained using 95% ethanol. The biofilm was determined based on visible disruption in biofilm formation and a significant reduction in the readings compared with the control wells at OD_570_ nm [30,32]. The cut-off OD for the microtiter-plate test is defined as three standard deviations above the mean OD of the negative control by following the classification according to Stepanovic et al. [25].
OD < ODc(non-adherent)ODc < OD ≤ 2ODc(weakly adherent)2ODc < OD ≤ 4ODc(moderately adherent)4ODc < OD(strongly adherent)

### 4.4. Antibiofilm Assay: Confocal Laser Scanning Microscopy (CLSM)

Confocal laser scanning microscopy (CLSM) was also used to confirm the antibiofilm activity of the fungal dextranase by observing cell attachment based on biofilm formation. *S. mutans* ATCC 25175 was used as a biofilm producer in this study. This experiment included fungal dextranase from TISTR 3511, the commercial fungal dextranase and the control (uninoculated medium without addition of dextranase). The experiment was a modification of the broth dilution method [30]; *S. mutans* was grown in TSB and adjusted to 10^6^ cells/mL; then, 1 mL *of S. mutans* cell suspension was mixed with 1 mL of cell-free culture (crude fungal dextranase). The biofilms were allowed to form on a 1 cm × 1 cm glass slide placed in 24-well titer plates, followed by incubation at 37 °C and 5% CO_2_ for 24 h. After 24 h incubation, the biofilm that was formed was stained with SYTO 9 green fluorescent dye (Thermo Fisher Scientific, Waltham, MA USA). The samples were assessed by Nikon Laser Confocal Microscope C1 (Nikon Instruments, Tokyo, Japan). Images were captured and processed using EZ-C1 version 3.90 (Nikon software, Tokyo, Japan). The biofilm images were observed using a 20× objective (20× N.A 0.75, W.D 1.00 Air), and images were acquired with 512 × 512 resolutions. Biofilm was analyzed in a series of X-Y images in which each image corresponded to a single Z position (depth), as shown in Figure 1.

### 4.5. Optimization of Dextranase Production from Fungus

Two steps were used to optimize dextranase production from fungus based on experimental designs, Plackett–Burman (PB) and Box–Behnken (BB). The production of dextranase enzyme by *P. roquefortii* TISTR 3511 was firstly optimized by the PB design to investigate the most significant fermentation parameters affecting the dextranase [29]. The variables chosen for the present study include medium components (dextran, yeast extract, K_2_HPO_4_, NaNO_3_, MgSO_4_.7H_2_O), incubation time, inoculum size, medium volume and inoculum age: Nine assigned variables in the PB design of 12 experimental runs. Each independent variable was tested at two levels, high and low. The experimental design, the name, the symbol code and the actual level of the variables are shown in Table 6.

According to the results of PB design from the previous experiment (Table 2), the significant factors (dextran, yeast extract and inoculum age) affecting dextranase production were varied to determine their optimal levels by using the BB design [33]. Thus, three variables were designed for three levels (low, medium, high) as shown: Dextran (20, 50, 80 g/L), yeast extract (10, 20, 30 g/L) and inoculum age (3, 5, 7 days). The 17 treatments were assigned variables in this experiment (details in Table 3). The software package Design Expert, version 10.0 (Stat-Ease Inc., Minneapolis, MN, USA) was used for the experimental design, data analysis and model building. Dextranase activity was measured using an increasing ratio of reducing sugar concentration as previously mentioned. Analysis of variances (ANOVA) was applied to assess the effects of studied variables, interactions, and statistical significance of models. The fitness of the polynomial model equations was expressed by the coefficient of determination R^2^ (Peng et al., 2020). Three-dimensional response surface plots were drawn to identify the interaction between factors and responses. The experiments for PB and BB designs were conducted in three biological replicates with two technical replicates.

### 4.6. Encapsulation of Fungal Dextranase Enzyme

The optimal conditions for fungal dextranase from *P. roquefortii* TISTR 3511 is close to that of commercial fungal dextranase of *C. gracile*; therefore, the model of fungal dextranase encapsulation used in commercial dextranase based on the BB design was adopted. The optimum conditions for encapsulation were set as pH of 7, 20% calcium chloride and 0.85% sodium alginate. Capsules prepared using the Buchi encapsulator B-390 were used for the encapsulation of dextranase. The crude dextranase from the strain TISTR 3511 solution and the 0.85% *w*/*v* sodium alginate solution were mixed at a ratio of 1:1, and the pH was adjusted to 7. The nozzle type and size used were single 450 µm, 160 Hz of frequency, 11–15 mL/min flow rate, 500–600 V for the electrode, 100–150 m Bar air pressure and 30 min hardening time. The capsules were dropped into a 20% calcium chloride solution, and the beads were filtered and washed twice with distilled water to eliminate excess calcium chloride [19]. The characteristics and mean diameter of beads were studied using ZEN lite imaging software of Stereo microscopes (ZEISS Stemi 305, Toronto, ON, Canada) according to the instructions.

### 4.7. The Activity of Dextranase Trapped in Alginate Beads

The beads at 1 g were grained with 1 g of 0.1 M phosphate buffer saline (PBS, pH 6.0); 125 µL of bead solution was mixed with 125 µL of dextran solution (20 mg/mL) for 30 min to allow the reaction to complete. Moreover, 250 µL of DNS reagent was pipetted into a test tube. All test tubes were immersed in a boiling water bath for 15 min. The reaction solution was pipetted into a microplate. The reaction was measured with a UV spectrophotometer at a wavelength of 540 nm. The activity of dextranase was calculated as previously described [19].

### 4.8. The Stability of Dextranase Activity in a Toothpaste Base

The toothpaste base is a composite of humectant, preservative, sweetening agent, abrasive, coloring agent and detergent [34]. On the basis of preliminary work, 2% (*w*/*w*) encapsulated dextranase alginate beads were added into the toothpaste base. Moreover, the dose selection was considered based on the range of human equivalent dose (HED) for clinical trial safety. The dose selection at 2% showed good physical characteristics. The guidelines of the Thai industrial standards (TIS 45-2552-2009) were followed to evaluate the stability of toothpaste storage. The toothpaste was subjected to accelerated storage conditions at 40 ± 2 °C for three months, and the stability of the encapsulated dextranase activity was further evaluated. Dextranase activity was measured using an increasing ratio of reducing sugar concentration in the reaction with 3,5-dinitrosalicylic acid reagent [1].

## 5. Conclusions

It can be concluded that *P. roquefortii* TISTR 3511 is a promising dextranase producer, while its crude dextranase acted against cariogenic *S. mutans* ATCC25175 to prevent biofilm formation. Dextran, yeast extract and age of inoculum are significant factors that enhanced dextranase production, and under optimum conditions a significant increase in dextranase activity, roughly four-fold, was observed. The stability of encapsulated dextranase in alginate beads supports the great potential of applying it in toothpaste base as a novel product for preventing dental caries and dental plaque.

## Figures and Tables

**Table 1:** The effect of crude dextranase from *Penicillium roquefortii* tistr 3511 on the biofilm-adherent capability of *Streptococcus mutans* atcc 25175.

Treatment	OD_570_ nm		Adherence Capability
	0 h	24 h	
Uninoculated medium (negative control)	0.24 ± 0.00	0.24 ± 0.00	non-adherent
*S. mutans* (positive control, no added dextranase)	0.25 ± 0.01	0.98 ± 0.01	strongly adherent
*S. mutans* in the presence of crude dextranase	0.24 ± 0.00	0.69 ± 0.00	moderately adherent

**Table 2:** Statistical analysis of factors affecting the amount of fungal dextranase enzyme.

Source	Sum of Squares	df	Mean Square	F Value	*p*-Value Prob > F
Model	524.72	9	58.30	58.76	0.017 *
A—dextran	391.18	1	391.18	394.27	0.003 *
B—yeast extract	53.87	1	53.87	54.29	0.018 *
C—K_2_HPO_4_	0.016	1	0.016	0.016	0.910
D—NaNO_3_	6.09	1	6.09	6.14	0.132
E—MgSO_4_.7H_2_O	0.23	1	0.23	0.23	0.679
F—Incubation time	9.19	1	9.19	9.26	0.093
G—Inoculum size	16.55	1	16.55	16.68	0.055
H—Medium volume	5.52	1	5.52	5.56	0.142
I—Inoculum age	42.07	1	42.07	42.40	0.023 *
Residual	1.98	2	0.99		
Corrected Total	526.70	11			

* Significance. The significance of the model (*p* = 0.017); the significant factors included dextran (*p* = 0.003), yeast extract (*p* = 0.018) and inoculum age (*p* = 0.023). Therefore, the most significant factors affecting dextranase production are dextran, yeast extract and inoculum age. The 12 experimental sets were suitably designed using Plackett–Burman (PB) design, and the ANOVA was fitted to the results as shown in the following equation.

**Table 3:** Use of Box–Behnken design to investigate the optimization conditions for producing dextranase enzyme (unit/g) from *P. roquefortii* TISTR 3511.

Condition	Significant Factor			Actual Value *	Predicted Value
	Dextran (g/L)	Yeast Extract (g/L)	Inoculum Age (Days)	Dextranase Activity (unit/g.)	Dextranase Activity (unit/g.)
1	50	30	7	29.57	29.31
2	20	30	5	25.87	26.16
3	50	20	5	28.46	28.26
4	50	10	7	25.67	25.34
5	50	20	5	28.45	28.26
6	50	30	3	27.68	28.01
7	80	10	5	25.46	25.17
8	80	20	7	28.12	28.74
9	80	20	3	26.35	26.38
10	20	20	3	24.87	24.25
11	50	20	5	27.47	28.26
12	50	20	5	28.46	28.26
13	50	10	3	23.57	23.83
14	80	30	5	30.24	29.88
15	20	10	5	22.35	22.71
16	20	20	7	24.78	24.71
17	50	20	5	28.47	28.26

* Dextranase activity was determined under its suitable condition at pH 6 and 37 °C.

**Table 4:** Statistical analysis of factors affecting the dextranase production with the Box–Behnken design experiment.

Source	Sum of SQUARES	df	Mean Square	F Value	*p*-Value Prob > F
Quadratic Model	73.49	9	8.17	24.40	<0.001 *
A—Dextran	19.03	1	19.03	56.86	<0.001 *
B—Yeast extract	33.25	1	33.25	99.34	<0.001 *
C—Inoculum age	3.96	1	3.96	11.84	0.011 *
AB	0.40	1	0.40	1.19	0.312
AC	0.90	1	0.90	2.70	0.145
BC	0.01	1	0.01	0.03	0.861
A^2^	8.76	1	8.76	26.16	0.001 *
B^2^	2.97	1	2.97	8.87	0.021 *
C^2^	2.69	1	2.69	8.05	0.025 *
Residual	2.34	7	0.33	-	-
Lack of Fit	1.56	3	0.52	2.65	0.185
Pure Error	0.78	4	0.20	-	-
Corrected Total	75.84	16	-	-	-

* Significance.

**Table 5:** The stability of encapsulated fungal dextranase based on its activity in alginate beads of toothpaste base.

Treatment	Dextranase Enzyme Activity (unit/g. Beads)		*p*-Value
	T0	T3	
Toothpaste base containing crude dextranase from *P. roquefortii* TISTR 3511 in beads	21.69 ± 1.72 ns	19.61 ± 0.57 ns	0.090

Notes: T0 = day 0; T3 = storage product at 40 ± 2 °C, 3 months. The differences of data were compared with the *t*-test statistic; ns = no significant difference.

**Table 6:** Plackett-Burman (PB) design for dextranase production by *P. roquefortii* TISTR 3511.

Factor	Name	Low Actual	High Actual
A	Dextran (g/L)	10.00	50.00
B	Yeast extract (g/L)	10.00	20.00
C	K_2_HPO_4_ (g/L)	0.50	1.00
D	NaNO_3_ (g/L)	1.00	2.00
E	MgSO_4_.7H_2_O (g/L)	0.50	1.00
F	Incubation time (days)	5.00	7.00
G	Inoculum size (ml)	1.00	2.00
H	Medium volume (ml)	10.00	20.00
I	Inoculum age (h)	48.00	72.00
K	Dummy1	−1.00	1.00
L	Dummy2	−1.00	1.00

**Figure A2:**
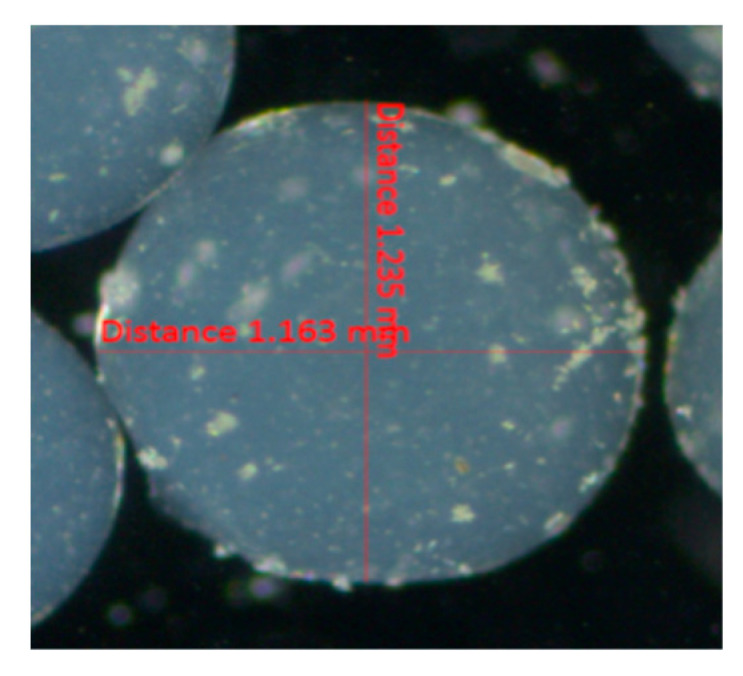
The regular spherical shape of alginate beads containing encapsulated fungal dextranase from *P. roquefortii* TISTR 3511 and diameter (mm).

**Figure A3:**
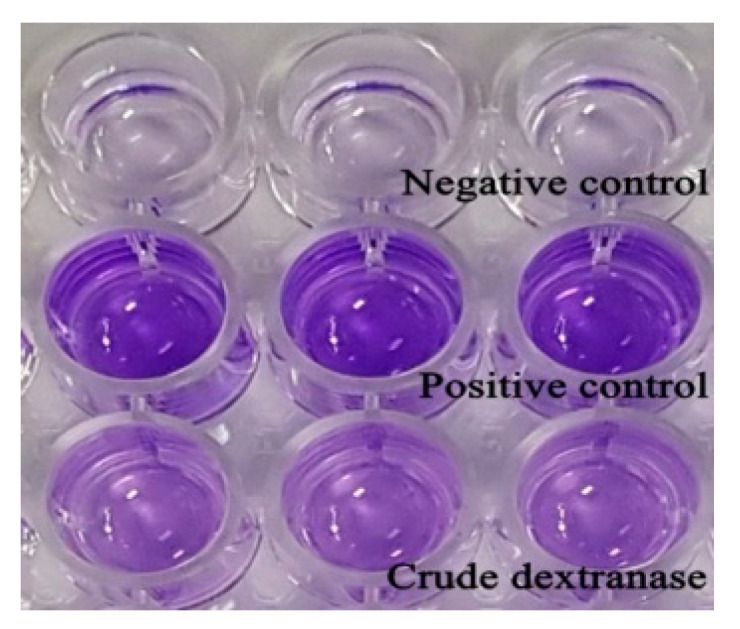
The effect of crude dextranase from *P. roquefortii* TISTR 3511 on biofilm adherent capability of *S. mutans* ATCC 25175, comparison between negative control, positive control and crude dextranase.
